# Successful implantation and removal of Impella 5.5 device via a 4-mm subclavian artery in an adult patient

**DOI:** 10.1007/s12055-024-01856-w

**Published:** 2024-11-25

**Authors:** Athanasios Tsiouris, Charles Mason Coleman, Ashok Kumar Coimbatore Jeyakumar

**Affiliations:** Department of Surgery, Division of Cardiac Surgery, University of Mississippi Medical Center, 2500 N State St, Jackson, MS 39216 USA

**Keywords:** Impella 5.5, Small axillary artery, Implantation, Removal, Heart transplant

## Abstract

Recently, the utilization of Impella 5.5, especially in the axillary position, has increased exponentially. The device provides excellent hemodynamic support for patients in cardiogenic shock, as a bridge to recover, transplant, or durable left ventricular device. However, small size arteries remain its main limitation. With a maximum diameter of 19 Fr (6.33 mm), the recommended artery size needed for implantation is 7 mm. In this case, we discuss the successful implantation and removal of the Impella 5.5 device into a patient, with a subclavian artery diameter of 4 mm, whose other short- and mid-term options for mechanical circulatory support were limited.

## Introduction

The Impella 5.5 with SmartAssist System was approved for short-term use in the setting of cardiogenic shock in 2019 [1]. Since its approval, it has grown in popularity, especially in the axillary position, due to its ease of insertion, excellent circulatory support, and the flexibility it allows patients in terms of their mobility [2]. However, small size axillary/subclavian arteries have been the main limiting factor in the usage of this percutaneous ventricular assist device (VAD) in pediatric and smaller adult population, with the recommended axillary artery size needed for implantation being 7 mm. The maximum diameter of the device is 19 Fr (6.33 mm) [3]. In this case, we discuss the successful implantation of the Impella 5.5 device into a patient with a subclavian artery diameter of 4 mm.

## Case report

**Fig. 1:**
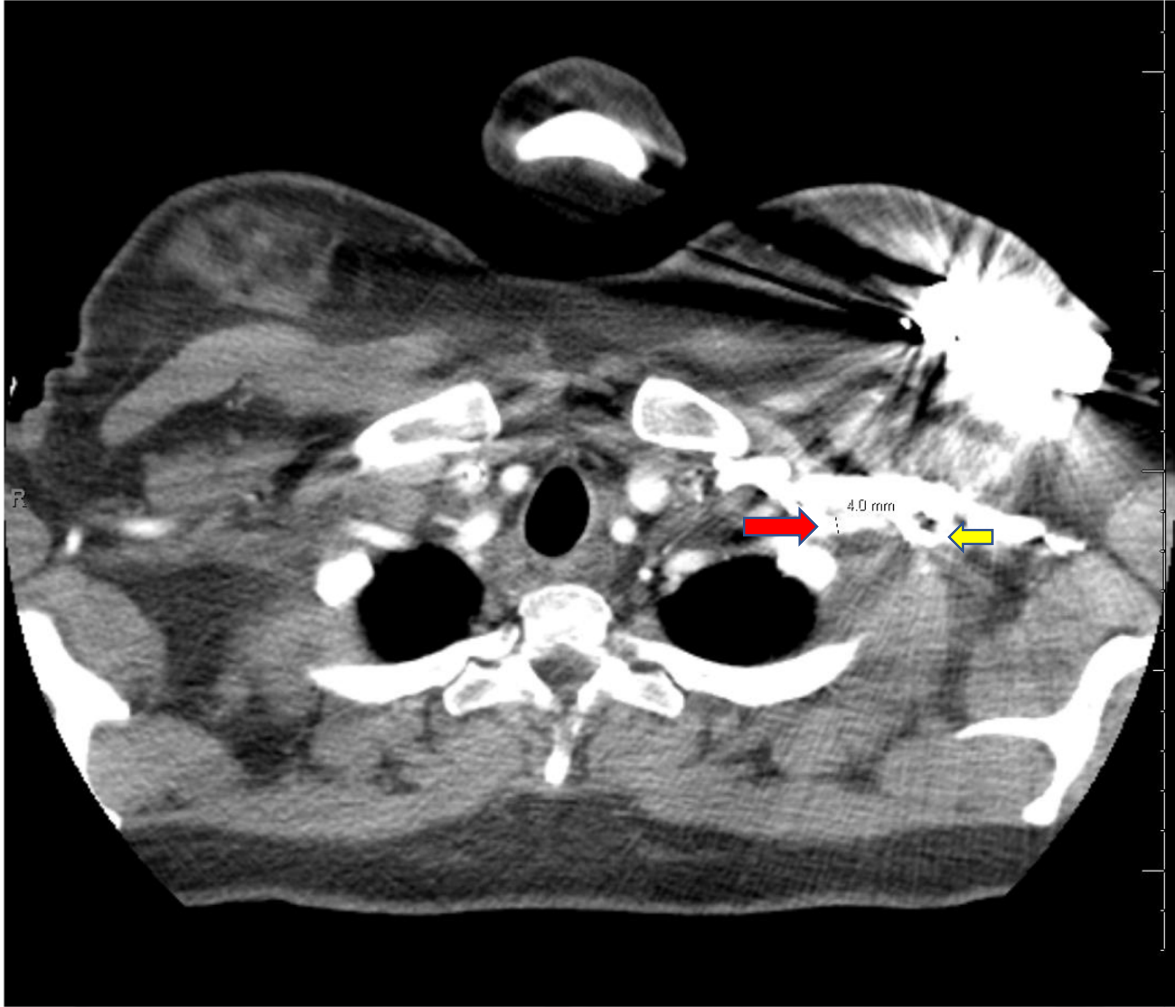
Four-millimeter left subclavian artery (red arrow). The left axillary artery distally is obscured by transvenous implantable cardioverter defibrillator (ICD) leads, but is visibly smaller (yellow arrow)

**Fig. 2:**
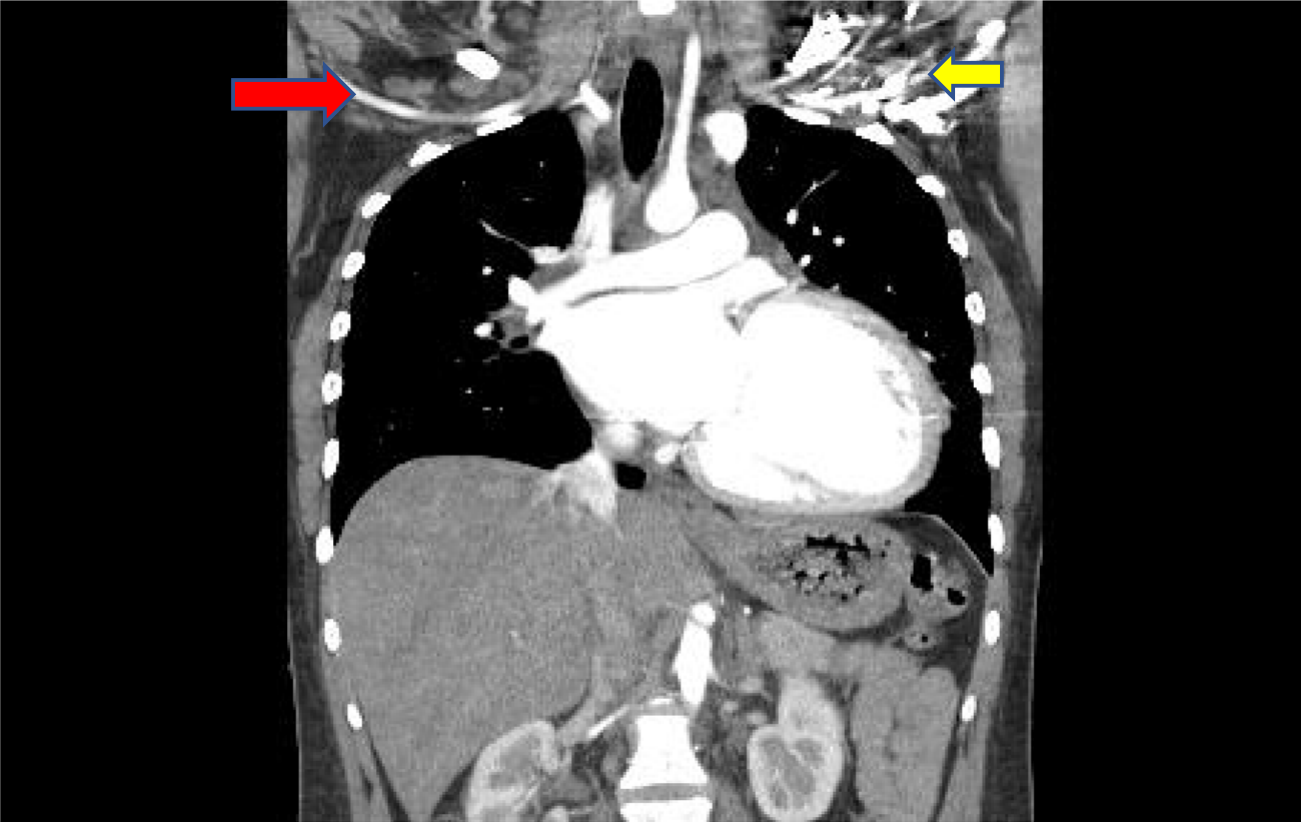
Four-millimeter left subclavian artery (yellow arrow). Smaller right axillary artery (red arrow)

The patient is a 30-year-old female with a history of peripartum cardiomyopathy 4 years ago, with an ejection fraction of 15–20%. She was referred to our institution a year prior after developing cardiogenic shock requiring high doses of inotropic support. After initial stabilization, she underwent a right heart catheterization (RHC) that demonstrated a cardiac index of 1.2 L/min/m^2^, a transpulmonary gradient of 15 mmHg, a wedge pressure of 25 mmHg, right atrial pressure of 12 mmHg, and pulmonary vascular resistance of 2.5–2.8 Wood units. Despite being on appropriate guideline-directed medical therapy (sacubitril-valsartan, spironolactone, dapagliflozin, bumetanide), she remained in class III symptoms and never showed evidence of left ventricular (LV) recovery. She was evaluated by our heart failure team and was deemed a good candidate for heart transplantation. She was placed on the transplant list at that time. She was once again admitted to our institution with cardiogenic shock and a repeat RHC demonstrated high left-sided filling pressures. A decision was made to pursue mechanical circulatory support in the form of an Impella 5.5 and uplist her to status 2 on the United Network Organ Sharing (UNOS) waitlist. A computed tomography (CT) chest (Fig. 1) demonstrated a left subclavian and axillary artery size of approximately 4 mm. The right axillary artery measured approximately 3 mm (Fig. 2). No calcifications were identified in the subclavian artery, aortic arch, or ascending aorta. She was ABO blood type O + , with a panel reactive antibody (PRA) of 15; her height was 1.46 m; and she weighed 57 kg. Given her body size, blood type, and antibody panel, we anticipated a potential long wait for an appropriate organ to become available. Despite the small size of her artery, a decision was made to attempt implantation of an axillary Impella 5.5, which would allow, during her wait for a suitable donor, continued ambulation, avoidance of physical deconditioning, and optimization of hemodynamics and fluid status prior to transplantation. Device longevity compared to other analogous forms of mechanical circulatory support also supported our decision to pursue an axillary Impella 5.5. Furthermore, we felt that an intra-aortic balloon pump (IABP) would not resuscitate or support her adequately.

**Fig. 3:**
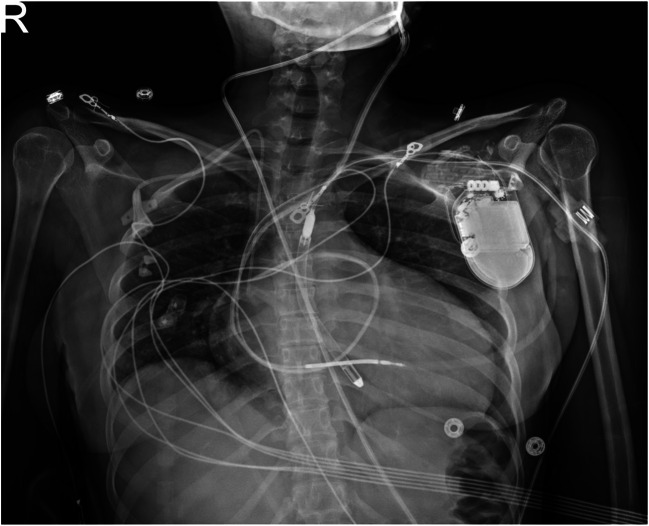
Chest x-ray with final placement of left axillary Impella 5.5

Access to the axillary artery was through the left deltopectoral groove. We encountered pre-existing implantable cardioverter defibrillator (ICD) leads which were retracted laterally. Subsequently, the left axillary artery was isolated using vessel loops. The vessel was then soaked in papaverine to promote vasodilation prior to arteriotomy. Vessels loops were then tightened proximally and distally and arteriotomy was performed and a 10-mm Hemashield graft was sewn. In order to mitigate expected resistance at the graft vessel junction, a steeper bevel of 50–60° was made on the 10-mm graft and the anastomosis was made wider than usual. The Impella 5.5 sheath was then inserted into the graft and under fluoroscopy, a 0.035 wire with a pigtail catheter was used to cross the aortic valve. A 0.18 stiff wire was placed through the pigtail and into the left ventricle. The Impella 5.5 was then advanced through the graft, axillary artery into the ascending aorta and into the left ventricle. Maneuvering the device through the anastomosis and the small subclavian artery occurred with ease, with a combination of gentle pressure, external manipulation of the axillary artery, and semirotational movement of the device, we were able to advance the Impella without complications. There was only mild resistance getting the device past the anastomosis, but advancing it through the artery was very easy. A maneuver we found extremely helpful was advancing the device by pushing it at the level of the graft and applying some countertraction of the axillary artery itself. This maneuver enhanced deliverability and also mitigated the torque response of the pump and device housing. In a standard fashion, the inlet of the Impella was placed 5 cm from the aortic valve, the peel away sheath was pulled, and the hub of the device was secured to the graft (Fig. 3).

Seventeen days later, the patient underwent orthotopic heart transplantation and removal of the pump. We decided to remove the distal portion of the Impella through the aorta, at the time of recipient cardiectomy, after dividing it with wire cutting scissors, just proximal to the pump motor. The remaining proximal housing portion of the device was retrieved from the axillary graft after temporary releasing the cross clamp. The axillary graft was clamped with a Fogarty clamp after the device was removed and trimmed down to 2–3 cm. The graft was then examined inside and thrombi were removed. The graft was allowed to bleed, distally and proximally. Large clips were placed on the graft which was also oversewn with a running 5–0 polypropylene suture. This short stump was left in the wound. We assessed the integrity of the upper extremity arterial flow by placing a pulse oximeter on the right hand and given that oxygen saturations correlated with the contralateral hand, we assumed no significant distal embolization had occurred. We also assessed for forward and back bleeding from the anastomosis, before ligating the graft, and we felt the pulse on the axillary artery distal to the anastomosis. At the end of the procedure, we also assessed the radial, ulnar, and palmar arch dopplers. The patient made an uneventful recovery and was discharged home on post-transplant day 10.

## Discussion

The Impella microaxial pump has emerged as a feasible alternative in stabilizing patients with hemodynamic collapse, as a bridge to recovery, transplant, or durable left ventricular assist device (LVAD) [4]. It can generate up to 6 L/min of cardiac output and can be inserted via transfemoral, trans-axillary, or trans-aortic approaches [3]. Trans-femoral Impella cardiac power (CP) insertions are complicated by peripheral vascular ischemia, while patients are forced to remain bedridden. The more popular axillary insertion for the Impella 5.5 can be limited by vessel size, while the direct aortic approach requires a sternotomy. Alternatively, trans-carotid insertions [5], through a cervical incision and trans-innominate artery approaches [6], via a supra-sternal/supra-clavicular incision have also been described, although both are limited by individual patient anatomy and also a theoretical high risk of stroke, mainly when accessing the carotid artery, which would be a devastating and unacceptable complication for potential heart transplant candidates.

Shugh et al. [7] published a successful implantation of an axillary Impella 5.5 device, as a bridge to recovery in an 11-year-old male with a 4.8-mm axillary artery. This is the only similar published case we identified in the literature. Our case demonstrates that with proper technique modifications, implantation of an Impella 5.5 in adults with even smaller axillary arteries is certainly possible.

## Conclusion

We describe a reproducible implantation technique for inserting Impella 5.5 VADs in adults with smaller arteries, without the need for an invasive central strategy. This is potentially extremely helpful for pediatric and smaller sized patients. In such situations, it is important to confirm the absence of atherosclerotic disease in the upper extremities, as calcium-free vessels are potentially distensible and will allow advancement of larger devices through them. We also recommend utilizing a steeper bevel on the graft; avoiding a small anastomosis; utilizing vasodilatory agents, such as papaverine; and applying counter tension directly onto the artery as forward pressure is applied onto the Impella at the level of the graft. We do strongly recommend aborting attempts to advance the Impella 5.5 in smaller arteries, if any type of resistance occurs. We also exemplify a successful method for removal of the device prior to orthotopic transplantation that does not damage the axillary or subclavian artery.

## References

[1] Ramzy D, Soltesz E, Anderson M. New surgical circulatory support system outcomes. ASAIO J. 2020;66:746–52. 32541335 10.1097/MAT.0000000000001194 PMC7316144

[2] Salter BS, Gross CR, Weiner MM, Dukkipati SR, Serrao GW, Moss N, et al. Temporary mechanical circulatory support devices: practical considerations for all stakeholders. Nat Rev Cardiol. 2023;20:263–77. 36357709 10.1038/s41569-022-00796-5 PMC9649020

[3] https://www.abiomed.com/products-and-services/impella/impella-55-with-smartassist. Last accessed May 2024.

[4] Gill G, Rowe G, Chen Q, Malas J, Thomas J, Peiris A, et al. Bridging with surgically placed microaxial left ventricular assist devices: a high-volume centre experience. Eur J Cardiothorac Surg. 2023;1;63. 10.1093/ejcts/ezad116 PMC10257579 36975609

[5] Akbar A, Tompkins B, Kilic A. A novel technique for microaxial left ventricular assist device insertion via transcervical transcarotid approach. JTCVS Tech. 2023;22:239–42. 38152202 10.1016/j.xjtc.2023.10.007 PMC10750980

[6] Bouhout I, Nguyen SN, Barry OM, Bacha EA, Goldstone AB. Transinnominate Impella 55 insertion as a bridge to transplantation in a pediatric patient in refractory cardiogenic shock. JTCVS Tech. 2022;14:201–3. 35967222 10.1016/j.xjtc.2022.06.005 PMC9367200

[7] Shugh S, Chrisant M, D'Addese L, Turner I, Bibevski S, Scholl F. Successful implantation of Impella 5.5 device and subsequent recovery in a pediatric patient with small arterial vessel. J Heart Lung Transplant. 2021;40:supplement, S523-S524.

